# Retraction: Interaction of fibrinogen and muramidase-released protein promotes the development of *Streptococcus suis* meningitis

**DOI:** 10.3389/fmicb.2026.1868466

**Published:** 2026-05-05

**Authors:** Frontiers Editorial Office

The journal retracts the article cited above.

Following publication, concerns were raised regarding the integrity of the images in the published figures.

The authors failed to provide a satisfactory explanation during the investigation, which was conducted in accordance with Frontiers' policies. As a result, the data and conclusions of the article have been deemed unreliable and the article has been retracted.

This retraction was approved by the Chief Editors of Frontiers in Microbiology and the Chief Executive Editor of Frontiers. The authors have not responded to correspondence regarding this retraction.

